# Characterization and Function of the First Antibiotic Isolated from a Vent Organism: The Extremophile Metazoan *Alvinella pompejana*

**DOI:** 10.1371/journal.pone.0095737

**Published:** 2014-04-28

**Authors:** Aurélie Tasiemski, Sascha Jung, Céline Boidin-Wichlacz, Didier Jollivet, Virginie Cuvillier-Hot, Florence Pradillon, Costantino Vetriani, Oliver Hecht, Frank D. Sönnichsen, Christoph Gelhaus, Chien-Wen Hung, Andreas Tholey, Matthias Leippe, Joachim Grötzinger, Françoise Gaill

**Affiliations:** 1 Université de Lille1-CNRS UMR8198, Laboratoire GEPV, Ecoimmunology of Marine Annelids (EMA), Villeneuve d'Ascq, France; 2 Institute of Biochemistry, Christian-Albrechts-Universität, Kiel, Germany; 3 Université Pierre et Marie Curie-CNRS UMR7144, Laboratoire AD2M, Adaptation et Biologie des Invertébrés en Conditions Extrêmes (ABICE), Station Biologique, Roscoff, France; 4 IFREMER, Centre de Brest, REM/EEP/LEP, Plouzané, France; 5 Department of Biochemistry and Microbiology and Institute of Marine and Coastal Sciences, Rutgers University, New Brunswick, New Jersey, United States of America; 6 Otto Diels Institute for Organic Chemistry, Christian-Albrechts-Universität, Kiel, Germany; 7 Institute of Zoology, Zoophysiology, Christian-Albrechts-Universität, Kiel, Germany; 8 Division of Systematic Proteome Research, Institute for Experimental Medicine, Christian-Albrechts-Universität, Kiel, Germany; 9 Université Pierre et Marie Curie-Muséum National d'Histoires Naturelles CNRS BOREA IRD, Paris, France; Cairo University, Egypt

## Abstract

The emblematic hydrothermal worm *Alvinella pompejana* is one of the most thermo tolerant animal known on Earth. It relies on a symbiotic association offering a unique opportunity to discover biochemical adaptations that allow animals to thrive in such a hostile habitat. Here, by studying the Pompeii worm, we report on the discovery of the first antibiotic peptide from a deep-sea organism, namely alvinellacin. After purification and peptide sequencing, both the gene and the peptide tertiary structures were elucidated. As epibionts are not cultivated so far and because of lethal decompression effects upon Alvinella sampling, we developed shipboard biological assays to demonstrate that in addition to act in the first line of defense against microbial invasion, alvinellacin shapes and controls the worm's epibiotic microflora. Our results provide insights into the nature of an abyssal antimicrobial peptide (AMP) and into the manner in which an extremophile eukaryote uses it to interact with the particular microbial community of the hydrothermal vent ecosystem. Unlike earlier studies done on hydrothermal vents that all focused on the microbial side of the symbiosis, our work gives a view of this interaction from the host side.

## Introduction

*Alvinella pompejana* is a polychaetous annelid that inhabits active deep-sea hydrothermal vents along the East Pacific Rise, where it colonizes the walls of actively venting high-temperature chimneys [1]. The environment of the worm is characterized by extreme physicochemical gradients, high pressure and bursts of elevated temperatures which can be as high as 105°C [2]. To date, the Pompeii worm is considered as one of the most eurythermal and thermotolerant metazoan known on Earth [3]–[6].

One of the striking features of this annelid is its association with a unique epibiotic bacterial community that forms cohesive hair-like projections from mucous glands lining the dorsal intersegmental spaces [3]. Numerous studies, including metagenomic analyses, evidenced that the microflora is composed of a multispecies complex of 12 to 15 phylotypes of which >98% are Epsilonproteobacteria, a dominating taxonomic group in hydrothermal vents [7]. These bacteria have been suggested to provide *Alvinella* with a stable source of nutrients and may detoxify the environment of the worm from reactive heavy metals and free hydrogen sulfide [1].

Central theme in beneficial bacterial-host interaction is that hosts must protect themselves against inappropriate colonization and replication of the symbiotic flora [8]. Various mechanisms are employed to control the symbionts without compromising host vitality. Amongst them, beneficial partnership between symbiotic bacteria and the immune reactions of the host has been widely invoked in mammals and insects [8]. The molecular interactions between the two partners of the association seem to modulate host immunity, and in turn the immune system shapes the composition of the microbiota.

Antimicrobial peptides (AMPs) are small sized molecules naturally produced by bacteria, protists, fungi, plants and animals. Their large distribution in nature within both unicellular and multicellular organisms suggests that they are crucial immune effectors which presumably have evolved under positive selection for a long period of time [9]. Recently, Login *et al* demonstrated that coleoptericin A, an AMP produced by the beetle belonging to the *Sitophilus* genus, keeps endosymbionts under control within the bacteriocytes [10]. By comparison with the number of AMPs isolated from terrestrial invertebrates (≈1500), relatively few AMPs (≈40) have been characterized from marine organisms [11]. Yet, marine animals are permanently in close contact with very high densities of microbes (10^5^ to 10^7^ per ml) suggesting that their immune effectors are effective in microbial growth inhibition and killing [12]. Although AMPs have been found in numerous marine invertebrate taxa such as Cnidarians, Annelids, Mollusks, Arthropods, Tunicates and Echinoderms [11], there is no evidence of active AMPs in organisms living in the deep-sea. To date, the aspect of AMP coevolution under selective pressures associated with the abyssal environment has never been investigated, whereas many life forms, in such an extreme habitat, rely on a symbiotic association.

Here, we describe the nature of the first abyssal AMP found in a symbiotic animal, its common origin with AMPs of coastal annelids as well as the manner in which an extremophile eukaryote uses it to interact with the particular microbial community of the hydrothermal vent ecosystem.

## Material and Methods

### Biological materials

#### Animal collection

*Alvinella pompejana* were collected from the bio9 and P Vent sites (EPR 9°50′N, 2.500 m depth) on board of the R/V L'Atalante using the telemanipulated arm of DSV Nautile (MESCAL Cruises 2010, 2012). Animals were brought back to the surface inside an insulated basket and directly dissected upon recovery. Although not subjected to specific property regulations (international water areas), authors have obtained permission to use samples for any analysis from both chief-scientists. This study did not involve endangered or protected species.

#### Primary cell culture

Freshly harvested coelomic cells were cultured in Leibovitz L-15 medium under sterile conditions on board. For microbial treatment, cells were separately incubated in 500 µL of medium containing 10 µL of killed bacteria, for 12 h. Incubations without bacteria were performed under the same conditions as controls.

#### Microorganisms

The bacterial strains used in this study are listed in S.1 in Material and Methods S1. Epibionts were scraped with a thin razor from 1 cm^2^ of the tegument of *Alvinella* freshly harvested and were suspended in 4 mL of sterile seawater. Primary enrichment cultures were obtained shipboard by adding an aliquot of epibionts or fragments of *Alvinella* tubes to 10 mL of modified SME media prepared as previously described and followed by incubation at 30 and 50°C [13], [14].

### Peptide purification and identification

A purification guided assay (see S.2 in Material and Methods S1 for details) was performed from the coelomic liquid of *Alvinella*. After three steps of chromatography (Reverse Phase-HPLC), the purity of the antimicrobial fractions was assessed by mass spectrometry (MS) analyses (DE STR PRO; Applied Biosystems) and homogeneous material was subjected to protein sequencing via Edman degradation (pulse liquid automatic peptide sequenator, Beckman Coulter).

### Three dimensional structures

#### NMR spectroscopy

(see S.3 in Material and Methods S1 for details). The renaturated alvinellacin was submitted to NMR measurements on a Bruker Avance III 800 MHz spectrometer. The chemical shift data were deposited in the University of Wisconsin Biological Magnetic Resonance Bank database under the accession number 18085. All spectra were processed with the program NMRPipe [15] and analyzed with the program NMRView [16]. Models of the three dimensional structures of capitellacin were generated using the solution NMR structure of alvinellacin as template. Structure calculations were performed using the program CYANA [17]. The 10 best structures were selected as the final structural ensemble and were deposited (PDB accession code 2LLR).

#### Assignment of disulfide bridges in alvinellacin by Mass Spectrometry

(see S.4 in Material and Methods S1 for details) Alvinellacin was incubated with the endoproteinase Lys-C. For sequential Lys-C and tryptic digestion, trypsin was added to the Lys-C digestion. During the incubation, aliquots were taken from the digest at different time points in order to monitor the enzyme digestion efficiency. MS experiments were performed on an offline nanoESI-LTQ Orbitrap Velos mass spectrometer with ETD option (Thermo Fisher Scientific, San Jose, CA). Spectrum interpretation and disulfide bridge assignment were performed manually.

### Alvinellacin activities

#### Antimicrobial assays

The minimal inhibitory concentration (MIC) and minimal bactericidal concentration (MBC) of the synthetic peptide (diluted in acidified water 0.05% acetic acid) against bacterial growth were determined by a microdilution susceptibility assay in microtiter plates as previously described [18]. Permeabilization of membranes of viable bacteria and pore-forming activity towards liposomes were measured as previously described in details [19], [20]. Alamethicin, cecropin P1, and magainin II were purchased as synthetic peptides from Sigma.

#### Shipboard antimicrobial assays against epibionts

Epibionts were scrapped with a thin razor from the tegument of freshly harvested *Alvinella*. They were incubated in presence or absence (control) of the alvinellacin peptide for 4 h and were subsequently fixed in 3% glutaraldedyde on board of the ship. In the laboratory, samples were directly placed on copper grids and counterstained with uranyl acetate and lead citrate. Morphological changes on epibionts were detected and damaged *versus* intact bacteria were counted on a Hitachi H 600 electron microscope.

### Gene characterization and gene expression

*The nucleotidic sequence* coding the preproalvinellacin precursor was obtained by blasting the amino acid sequence of Alvinellacin to the *Alvinella* EST database (TERA 00513) [21].

#### Gene structure

The complete gene sequence of the preproalvinellacin (Genbank accession number KJ489380) was obtained from the cloning of PCR-products coming from the nested amplification of a series of *A. pompejana* gDNA using specific primers targeted on the 5′ and 3′ ends of the cDNA. Used primers are as followed: AP_alvinellacinF starting from the first methionine codon: 5′-ATG ACG TAT TCT GTA GTT GTG ACG CTG GTC-3′, AP_alvinellacinR1 (in the 3′UTR region): 5′-TAG GCA GGA CGG AGC CGC CAG ATC A-3′, and AP_alvinellacinR2 (starting on codon stop): 5′-CTC AGT GAA ATG AAG CAG GTG AGT TAT G-3′. PCR amplifications were obtained following 40 cycles of 96°C for 45 s, 60°C for 45 s and 72°C for 4 min after a first denaturation of gDNA at 96°C for 4 min and a final elongation of 10 min. Putative splicing sites (ACEs and ISEs) and both mobile and regulatory elements were detected using ACESCAN2 web server (http://genes.mit.edu/acescan2/index.html) and modules of the geneinfinity (http://www.geneinfinity.org/sp/sp_coding.html), webgene (http://www.itb.cnr.it/webgene/) and the TE tools (ergmanlab.smith.man.ac.uk/?page_id = 295) platforms. The complete gene sequence of preprocapitellacin was obtained by blasting the preproalvinellacin in the *Capitella teleta* genome database (http://genome.jgi-psf.org/Capca1/Capca1.home.html).

#### Quantitative Reverse Transcription PCR

RNA from cells were extracted (Qiazol, Qiagen) and used for cDNA synthesis with an oligodT according to the protocol of the manufacturer (SuperScript II; Invitrogen). The primers used for quantification were designed with the Primer3 Input software (http://frodo.wi.mit.edu/cgi-bin/primer3/primer3 www.cgi).

-Alvinellacin primers: forward: 5′-TGACATCGTGAAGGAACTCG-3′; reverse: 5′-CCGTTCCTACCAACTTTCCA-3′

-Ribosomal Protein 26S primers (*Alvinella* database [21]: TERA01523): forward: 5′-CCGGCTAGTTCAAGATGACC-3′; reverse: 5′-AGCTGCTGCCTCCACTATGT-3′.

The RP26S was used as the reference gene. Real Time reactions were conducted on a CFX96 qPCR system (BioRad) using a hot start, then 40 cycles at 94°C, 15 s; 56°C, 30 s; 72°C, 30 s., and a final extension step at 72°C for 3 min. Analysis of relative gene expression data was performed using the ΔΔCt method. For each couple of primers, a plot of the log cDNA dilution versus ΔCt was generated to validate the qPCR experiments (data not shown). Reference and target were amplified in separated wells.

### Alvinellacin production sites

#### Polyclonal antiserum

The alvinellacin antiserum was raised in two New Zealand White rabbits (Saprophyte pathogen-free). The chemically synthesized peptide was coupled to OVA and used for the immunization procedure according to the protocol described previously [22]. The reactivity of the antibody was tested by Dot Immunobinding Assay (DIA) using 1 µL of the RP HPLC fractions [23].

#### Immunocytochemistry and immunohistochemistry

Cells or tissues were fixed on board in 4% paraformaldehyde. Later, the SHANDON Cytospin 3 was used to spin cell suspension onto poly-lysine slides (8 min, 2,000 rpm). Immunocytochemistry and immunohistochemistry were performed with the rabbit anti-alvinellacin (1∶100) and the FITC-conjugated anti-rabbit secondary antibody (1∶100; Jackson Immunoresearch Laboratories) according to a protocol already described by our group [21]. Samples were examined using a confocal microscope (Zeiss LSM 510).

#### Coelomocyte structure

The coelomocytes were collected, and immediately fixed in 3% glutaraldehyde according to the protocol previously described [24]. Coelomocytes were observed on a Hitachi H 600 electron microscope.

## Results and Discussion

### Nature of *Alvinella* AMP and evolutionary link with AMPs from coastal species

**Figure 1:**
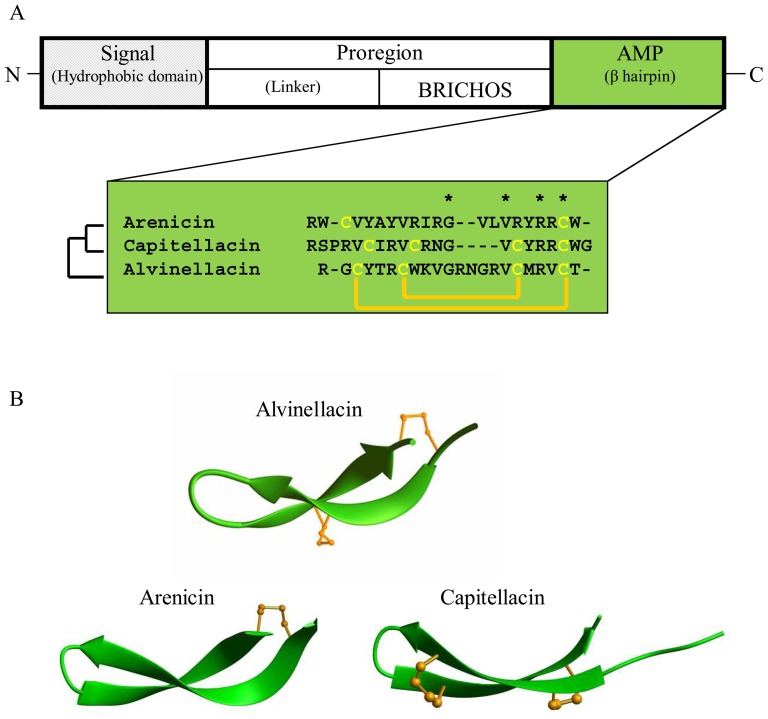
Alvinellacin is evolutionary linked to a family of AMPs from coastal annelids. (A) Molecular organization of the alvinellacin precursor and sequence alignment of alvinellacin with arenicin, an AMP produced by *Arenicola marina*, and capitellacin, a sequence so named by our group, issued from the analysis of the *Capitella teleta* genome. N and C denote N- and C-termini. (B) Comparison of the three-dimensional structures of the three AMPs. Disulfide bonds are depicted as yellow balls and sticks.

To date, only one AMP isolated from marine species belongs to an AMP family already characterized in terrestrial species [11]. The majority of marine AMPs presents novel structures and is confined to certain taxa or even species, as observed for AMPs of polychaeta [25]. In order to identify peptides with antibiotic activity from the Pompeii worm, a biochemical approach was combined with the analysis of the *Alvinella* EST database [22]. The anatomy of annelids is characterized by the presence of a coelom, a compartment that includes mobile cells, named coelomocytes that sterilize the coelomic fluid by releasing humoral factors such as antimicrobial peptides (AMPs) [26]. We purified and identified a cationic peptide composed of 22 amino-acid residues which we named alvinellacin, from the coelomocytes of *Alvinella* (Figures S1 and S2). As for most AMPs from all invertebrate phyla, mature alvinellacin is processed from a larger precursor molecule containing a signal peptide and an anionic proregion [27] (Figure 1A).

Following a BLAST search, the mature peptide did not display any similarity in its primary structure with other known proteins. However, the proregion had ∼33% identity to the proregion of AMPs from coastal annelids: arenicin from *Arenicola marina* and capitellacin, a putative peptide inferred from the genome sequence of *Capitella teleta* (Figure S3). Pfam analysis of the proregions revealed the presence of a conserved BRICHOS domain. So far, this 100 amino acids domain has never been reported in other AMP precursors than preproarenicin [28].

Despite the lack of an obvious similarity between the primary structures of alvinellacin, arenicin and capitellacin, we analyzed and compared their three-dimensional structures. Using NMR spectroscopy and mass spectrometry, we determined the tertiary structure of alvinellacin (Figures S4 and S5, Tables S1 and S2) and compared it to the solved and predicted structures of arenicin and capitellacin, respectively (Figure 1B). Alvinellacin and capitellacin are stabilized by two disulfide bonds, whereas arenicin possesses only one cystine. Like capitellacin, alvinellacin folds into a double-stranded antiparallel beta-sheet resembling the structure of arenicin [29]. Consequently, the three AMP precursors *i.e*. preproalvinellacin, preprocapitellacin and preproarenicin harbor the conserved pattern of almost all the BRICHOS containing proteins: a hydrophobic domain (here, the signal peptide), a linker region, the BRICHOS domain itself and a C terminal region with β-sheet propensities (here, the AMP) (Figure 1A) [28].

To date, proregions of AMP precursor are essentially known to be implicated in cell chemotaxy and/or protection against the cytotoxic activities of certain AMPs [30]. The BRICHOS domain has been found as a constituent of proteins associated with a wide variety of human diseases such as dementia, respiratory distress and cancer [31].

Recent data evidence that BRICHOS participates in the complex post-translational processing of proteins, and functions as an intramolecular chaperone domain that can bind β hairpin motifs and prevents them from β sheet aggregation and amyloid fibril formation [28]. Because of their strand-loop-strand structure, it seems reasonable that alvinellacin like the two other AMPs interacts with BRICHOS. Coastal and, even more, hydrothermal annelids are naturally submitted to strong hypoxic and thermal stresses. We hypothesize that the presence of the BRICHOS domain might be an evolution-driven adaptation of the worms to warrant the correct folding of their AMP under extreme conditions such as hypoxia and/or eurythermality. All these suggestions should be experimentally tested: BRICHOS might also have a novel function in *A. pompejana* that remains undiscovered.

As a conserved gene structure constitutes a convincing evidence for evolutionary relatedness between protein families, we also characterized the complete gene sequence of alvinellacin and compared it to the capitellacin gene [32] (Figure S6). Both genes display a 5 introns/6 exons structure with nearly all conserved intron-splicing positions. Given the taxonomic position of *Capitella* and *Alvinella* [33], their gene structure along with the proregion sequence identity and the three-dimensional peptide structure, strongly indicate that alvinellacin and capitellacin presumably together with arenicin, share an ancient origin and are evolutionary correlated since hundred millions of years. Further detailed comparisons of the proregions showed a high level of amino acid changes in the first part of the propieces; that may be also attributable to an adaptive ‘hot spot’ of mutations (functional change) in the face of the very long period of time since divergence between the two polychaeta species. The low amino acid conservation in the AMP sequences compared to the proregions suggests that they might have evolved independently. To the best of our knowledge, AMP proregions are not known to interfere with components of the external environment as AMPs do by interacting with microbes. Thus, the mature AMP presumably evolved to respond to the specific microbial communities (hydrothermal or coastal habitats) as well as to the specific lifestyle (symbiotic or not) of the worms while the proregion did not. This is consistent with the observation that the C-terminal propeptides of the interstitial collagen of *Alvinella* and *Arenicola* are similar, while the helical domain of the mature protein, which is located in the extracellular matrix and is presumably more exposed to environmental conditions, is not. [34].

### Alvinellacin in the first line of defense towards environmental microbes

**Figure 2:**
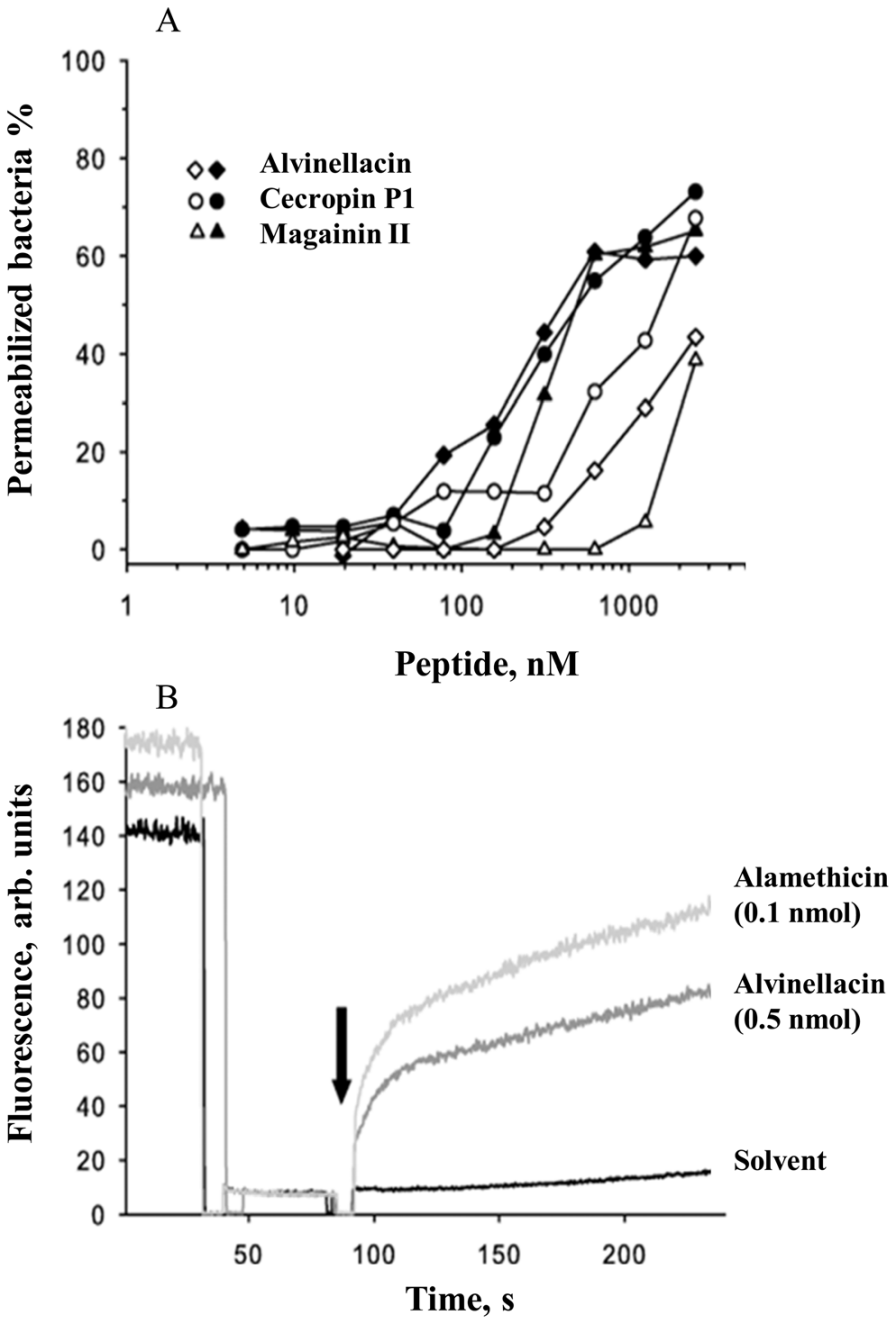
Bactericidal activity of alvinellacin. (A) Membrane permeabilization of viable bacteria induced by alvinellacin compared with that induced by other antimicrobial peptides. The percentage of bacteria with compromised membranes was monitored fluorometrically using the membrane-impermeable SYTOX green dye. Bacteria were incubated with varying concentrations of alvinellacin, cecropin P1 and magainin II at pH 7.4. Induction of membrane permeabilization was monitored against *B. megaterium* after 10 min (closed symbols) and against *E. coli* after 2 h (open symbols). (B) Time course of pore formation induced by alvinellacin. The dissipation of a valinomycin-induced diffusion potential in vesicles of asolectin after addition of alvinellacin (0.5 nmol), control peptide alamethicin (0.1 nmol), and the peptide solvent (0.05% acetic acid) were recorded. Pore-forming activity is reflected by the increase of fluorescence as a function of time. The arrow marks the time point at which the peptide is added.

In general, microbial invasion into the host causes bacterial infection which prompts an immune response such as the release of AMPs to eliminate invaders. Since alvinellacin was isolated from the coelomocytes, these cells are likely to produce and secrete the AMP into the coelomic fluid where it exerts its antibacterial activities. The presence of a signal peptide in the alvinellacin precursor (Figure 1A) together with the results obtained by immunocytochemistry (see below) corroborates this assumption. The antimicrobial activity of alvinellacin was then evaluated (Figure 2 and Table 1). As the worm's coelomic fluid composition is not very different from seawater, assays were performed at salt concentrations mimicking this environment [35]. Under these conditions, alvinellacin's activity was constant primarily against Gram-negative bacteria. This may represent an adaptation of the worm to its associated microorganisms, which have been shown to be predominantly Gram-negative ε-proteobacteria [3], [36]. We then wondered whether an exposure to various microorganisms might have differential impacts on the synthesis of alvinellacin. Usually, to investigate the immune response of an organism, animals are submitted to experimental infections and variations of immune markers are quantified. Since *Alvinella* precludes *in vivo* investigation because of lethal decompression effects upon sampling [6], [37], we developed an *ex vivo* model by establishing primary culture of cells obtained from freshly harvested animals.

**Table 1:** Antimicrobial activity of alvinellacin.

	MIC, µM	MBC, µM
**Gram-negative bacteria**		
*Escherichia coli D31*	0.012–0.024	0.048
*Escherichia coli D31* (300 mM NaCl)	0.012–0.024	0.048
*Escherichia coli D31* (500 mM NaCl)	0.012–0.024	0.048
Pseudomonas sp.*	0.001–0.003	0.012
Vibrio diabolicus*	0.048–0.096	>0.19
*Vibrio MPV19*	0.012–0.024	0.024
**Gram-positive bacteria**		
*Bacillus megaterium*	0.012–0.024	0.024
*Bacillus megaterium* (300 mM NaCl)	0.024–0.048	0.048
*Bacillus megaterium* (500 mM NaCl)	0.048–0.096	0.096
*Staphylococcus aureus*	0.048–0.096	>0.19

Assays were performed against bacteria routinely used for antimicrobial assays or having a medical incidence, and against the scarce hydrothermal strains (asterisk*) cultivable under the conditions of a microbial assay. The minimal inhibitory concentration (MIC) and the minimal bactericidal concentration (MBC) are expressed as final concentration in µM. > denotes no activity detected at the given concentration. The MBC and MIC values are the same, indicating that the bacterial growth inhibition is due to the killing of bacteria.

**Figure 3:**
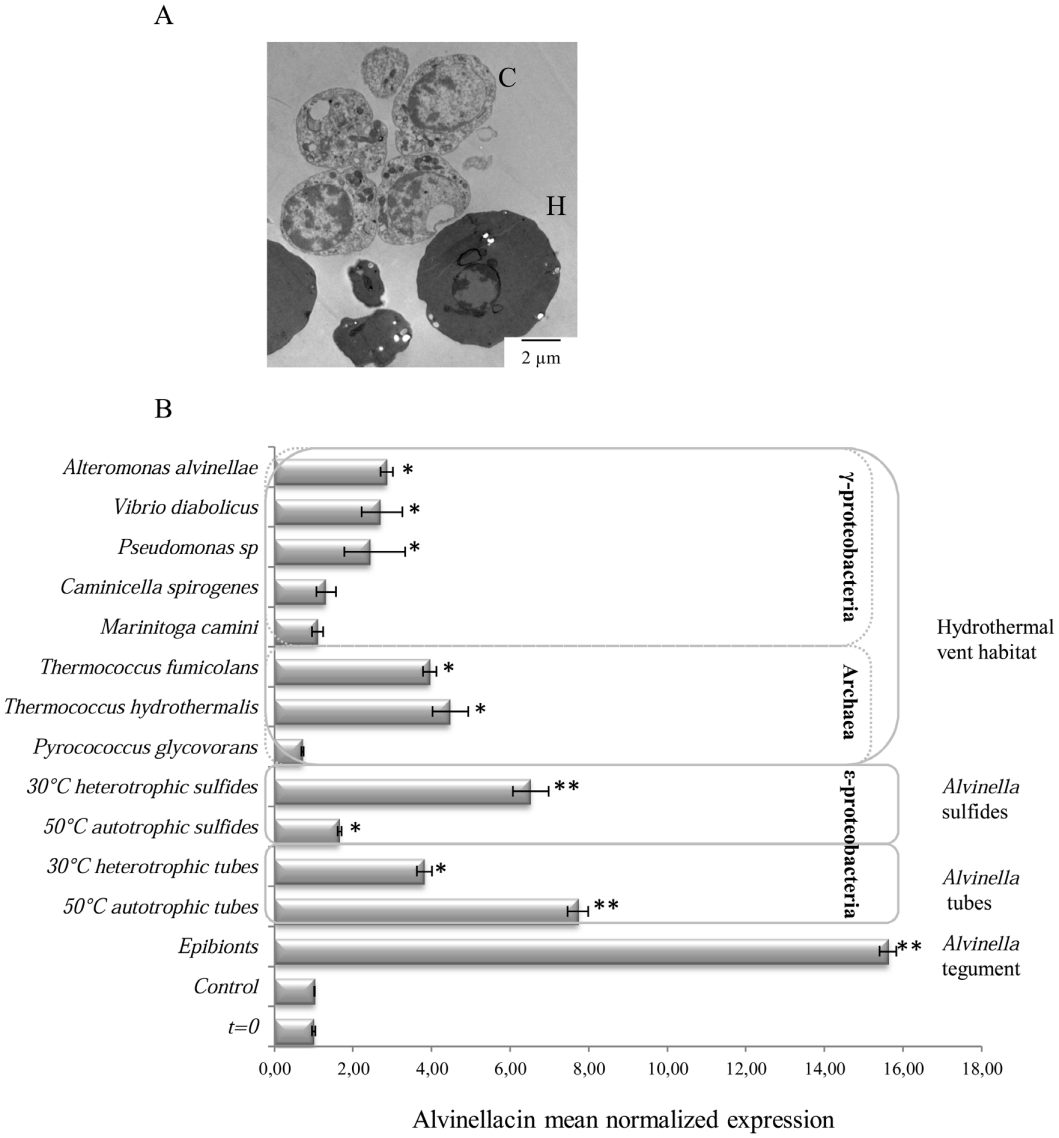
Selective induction of the gene encoding alvinellacin in cœlomocytes exposed to vent bacteria. (A) Electron-microscopic image showing the intact structure of *Alvinella* cœlomocytes despite 250 bars decompression. C: cœlomocytes. H: blood cells. (B) RT-qPCR on primary culture of coelomocytes incubated in the presence (t = 12 h) or not (control) of *Alvinella* epibionts, enrichment cultures obtained from *Alvinella* samples, and different pure cultures of bacteria and archaea isolated from hydrothermal vents. Graphics represent the results of two independent experiments; p-values from Student's tests were calculated *versus* the control treatment, based on the experimental measures performed in triplicates (*p<0.05).

Despite being subjected to 250 bars decompression, cells were alive and morphologically preserved (Figure 3A). Primary cell cultures were then initiated and maintained on board. To mimic a systemic infection, coelomocytes were incubated in presence of either *Alvinella* epibionts or of different vent bacterial strains (Figure 3B). Quantitative RT-PCR experiments showed a selective induction of the gene encoding alvinellacin upon exposure of the worm to the vent bacteria. Both epibionts, which are highly represented by ε-proteobacteria [7], and bacterial enrichments obtained shipboard from *Alvinella* tubes using culture conditions that support growth of vent ε-proteobacteria, appear to be better inducers than pure cultures of γ-proteobacteria. These results show that *Alvinella* coelomocytes can sense different microorganisms and that alvinellacin synthesis might be the outcome of the adaptation of *Alvinella*'s immune defense system against the specific microorganisms present in its environment. To date, the role of archaea in activating the host's immune system and the ability of its immune receptors to detect their presence has never been investigated. Interestingly, *Alvinella* tubes are inhabited by an extremely dense population of archaea related to the Thermococcales, including members of the two major genera, *Thermococcus* and *Pyrococcus*, the former being more prevalent in *Alvinella* tubes than the latter [38], [39]. We investigated the ability of these hyperthermophilic microbes to induce the expression of the alvinellacin gene in our cell cultures. Remarkably, only archaea belonging to the *Thermococcus* genus induced the expression of the alvinellacin gene. These data indicate that *Alvinella* can selectively recognize specific archaea and in turn induces an immune response, suggesting for the first time the existence of pattern recognition receptors in an eukaryote organism able to recognize and discriminate archaeal microbe-associated molecular patterns [40].

### Alvinellacin controls and shapes the epibiotic flora

**Figure 4:**
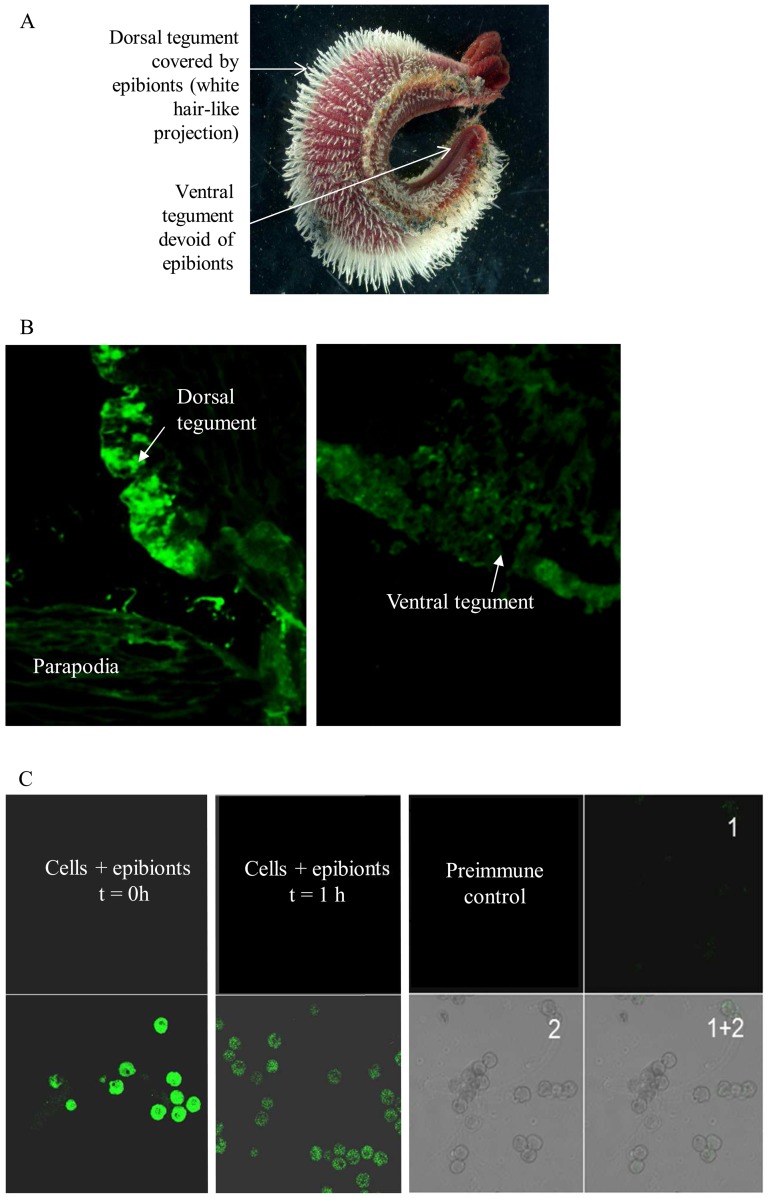
Alvinellacin is produced by tissues or cells in contact with epibionts. (A) Picture of *Alvinella* showing the distribution of epibionts. (B) Immunohistochemistry data evidence that alvinellacin peptide accumulates in tissue hosting epibionts *i.e*. the dorsal but not the ventral tegument. (C) Accidental entrance of epibionts stimulates the secretion of alvinellacin by circulating cells. Images of immunodetection of alvinellacin in coelomocytes incubated with epibionts. After one hour of exposure, the signal was reduced evidencing an extracellular secretion of the peptide. Control is performed with preimmune serum 1: FITC fluorescence, 2: transmission, 1+2: overlay.

**Figure 5:**
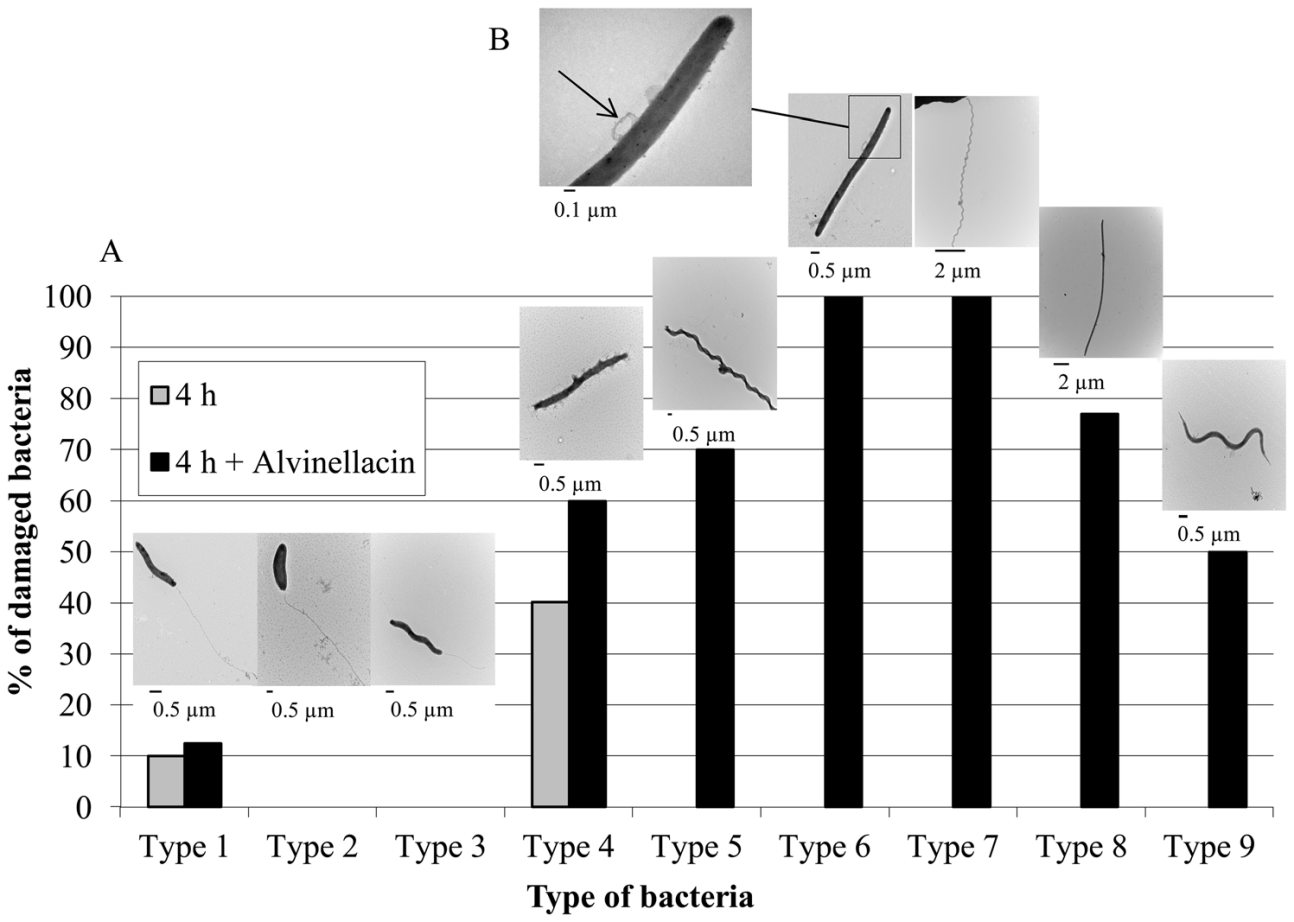
Alvinellacin activities against epibionts. (A) Freshly collected epibionts were incubated alone or in the presence of alvinellacin for 4 h, and after fixation observed under the electron microscope. The number of damaged bacteria was estimated among nine different morphotypes (numbered from 1 to 9) clearly distinguishable in all our preparations. (B) Bacterial lesions are visible at high magnification as the formation of membrane blebs and the release of cytoplasmic material (arrow).

While a key role of AMPs in fighting infections is well described, very recent studies also evidenced that these effector molecules can be employed to regulate/control the symbiotic microflora [10], [30]. The strong and vital relationship between *Alvinella* and its epibionts prompted us to investigate such an alternative function of alvinellacin. Immunohistochemistry experiments showed that alvinellacin is expressed constitutively by epithelial cells of the tegument associated with the epibiotic microflora, i.e. the dorsal but not the ventral epidermis (Figures 4A *vs* 4B). This observation supports the idea that alvinellacin may prevent bacterial entrance and/or keep epibionts under control. Accordingly, we determined the antimicrobial potency of alvinellacin against epibionts (Figure 5). As epibionts have not been cultivated so far, we carried out a shipboard antimicrobial assay aimed at detecting epibiont-cell damage in response to exposure to alvinellacin. Interestingly, alvinellacin significantly targeted epibionts that correspond to filamentous bacteria (epibiont types 5 to 9). In particular, alvinellacin killed 100% of the two most abundant morphotypes within this group (types 6 and 7). In contrast, the presence/absence of alvinellacin on epibionts types 1 and 4 did not have distinguishable effects and other epibionts (types 2 and 3) are not affected by alvinellacin. Altogether, the data suggest that alvinellacin controls epibiosis by selectively killing the most dominant part of the filamentous bacteria found on the dorsal part of the worm.

That is reminiscent of the role of the defensin HD5 in shaping the composition of the symbiotic microflora of the digestive tract in mammals [41]. We hypothesize that high production of alvinellacin by epidermal cells selects and shapes the epibiotic microflora and prevents microbiota from over proliferating and subsequently penetrating the underlying tissue. To test this hypothesis, an accidental invasion was simulated by incubating coelomocytes with epibionts (Figure 4C). The distribution of alvinellacin-immune reactivity was compared by immunofluorescence in unchallenged *versus* challenged cells. Under basal conditions (t = 0), the AMP was strongly detectable inside the cells, suggesting that this active compound is stored after synthesis. One hour after the bacterial infestation, the immune staining inside the cells faded, evidencing that alvinellacin is secreted rapidly when the cells are challenged by microorganisms. The induction of transcription observed by RT-qPCR in the cells incubated for 12 h with epibionts probably contributes to the renewal of the alvinellacin peptide stock (Figure 3B). Overall, these results reinforce the role of alvinellacin in keeping symbionts under control.

## Conclusion

Altogether, the data indicate the production of an original AMP from a deep-sea animal that endorses a durable relationship with Epsilonproteobacteria and possibly archaea in the face of the hostile vent habitat. Alvinellacin appears to act as a first line of defence against microbial invasion. The specificity of the gene induction along with the selective anti epibiotic activity and the expression in tissues exposed to the environment suggest that alvinellacin is actively participating in the surveillance of the epibiotic community. The conservation of the proregion and the gene structure of alvinellacin with AMPs of coastal annelids, suggest a common origin of the molecules. To draw a decisive conclusion regarding the gene evolution of alvinellacin, we plan to search for related genes in more than 30 annelid species living in various habitats. Such phylogenetic analysis will aim at determining whether the amino acid sequences of the antimicrobial part of the precursor diverged between species in order to face (i) contrasted temperatures, (ii) different microbial environments and/or (iii) to allow the establishment of epibioses.

## Supporting Information

Figure S1 **Alvinellacin purification and molecular identification.** Material eluting at 60% acetonitrile (ACN) upon solid phase extraction was loaded onto a C18 column (250×4 mm, Vydac). Elution was performed with a linear gradient of acetonitrile in acidified water (dotted line), and absorbance was monitored at 225 nm. Each individually collected fraction was tested for its antimicrobial activity (white bar) and its immunoreactivity to the alvinellacin Ab by DIA (grey bar). Fractions containing antimicrobially active alvinellacin were further purified by two additional RP-HPLC purification steps. Asterisk shows the active final fraction containing alvinellacin.
(TIF)

Figure S2 **MS spectrum of native alvinellacin.** Analysis of purified alvinellacin by MALDI TOF-MS shows a m/z value of 2,600.35 MH+ which perfectly matches the theoretical mass of the peptide including two disulfide bonds.
(TIF)

Figure S3 **Sequence alignments of the precursors of alvinellacin, capitellacin, and two arenicin isoforms.**
(TIF)

Figure S4 **Intact protein MS spectrum of alvinellacin measured by nanoESI-Orbitrap MS.** (A) Full range MS survey spectrum. (B) Zoom-in of the [M+5H]5+ charge state species in a. A small species (indicated as asterisk) found next to the major component was identified as the methionine oxidation product of alvinellacin. The experimentally determined monoisotopic MW of alvinellacin was 2,599.2221 Da. (C) Display of theoretical MW (2,599.2067 Da) of alvinellacin and its isotope distribution at charge state 5. The results indicated that all four cysteines are involved in the formation of disulfide bonds.
(TIF)

Figure S5 **Time-course analysis of the proteolytic cleavage of alvinellacin.** The products of alvinellacin digestion were analyzed by nanoESI-Orbitrap MS. (A) Peptide MS survey spectra of alvinellacin digested with Lys-C at 35°C (overnight). (B) Subsequent digestion of the Lys-C-digest with trypsin after 30 min; (C) after 2 h; (D) after 18 h at 37°C. The identities of the peptides are summarized in Table S2.
(TIF)

Figure S6 **Alvinellacin and capitellacin gene structures.** (A) As opposed to CDS (648 bp), the alvinellacin gene is rather long (1949 bp from the initial methionine to the stop codon) with a 5 introns/6 exons structure and a first large intron of 442 bp. Introns are all inserted in phase 0 with the exception of the last one in phase 1. (B) Alignment of the translated regions of the alvinellacin and capitellacin genes. The intron splicing positions (triangles) are nearly conserved. The BRICHOS domains are shaded and the AMP sequences are in bold type.
(TIF)

Material and Methods S1 (DOCX)

Table S1 **Structural statistics for the 10 best structures of alvinellacin showing the lowest target functions.** None of the distance constraints was violated by more than 0.5 Å in any structure.
(DOCX)

Table S2 **Disulfide-connected peptide fragments of alvinellacin observed after proteolytic cleavage.** Peptides with oxidized cysteines were successively digested using the proteases Lys-C and trypsin. The resulting peptides were analyzed by offline nanoESI-Orbitrap MS/MS as shown in Figure S2. The results unambiguously indicated two disulfide linkages between C1–C4 and C2–C3.
(DOCX)
