# Supplementary figures and images for: Characterization and Function of the First Antibiotic Isolated from a Vent Organism: The Extremophile Metazoan Alvinella pompejana
Source: PLoS One. 2014 Apr 28;9(4):e95737. doi: 10.1371/journal.pone.0095737 (PMC4002450; doi:10.1371/journal.pone.0095737)

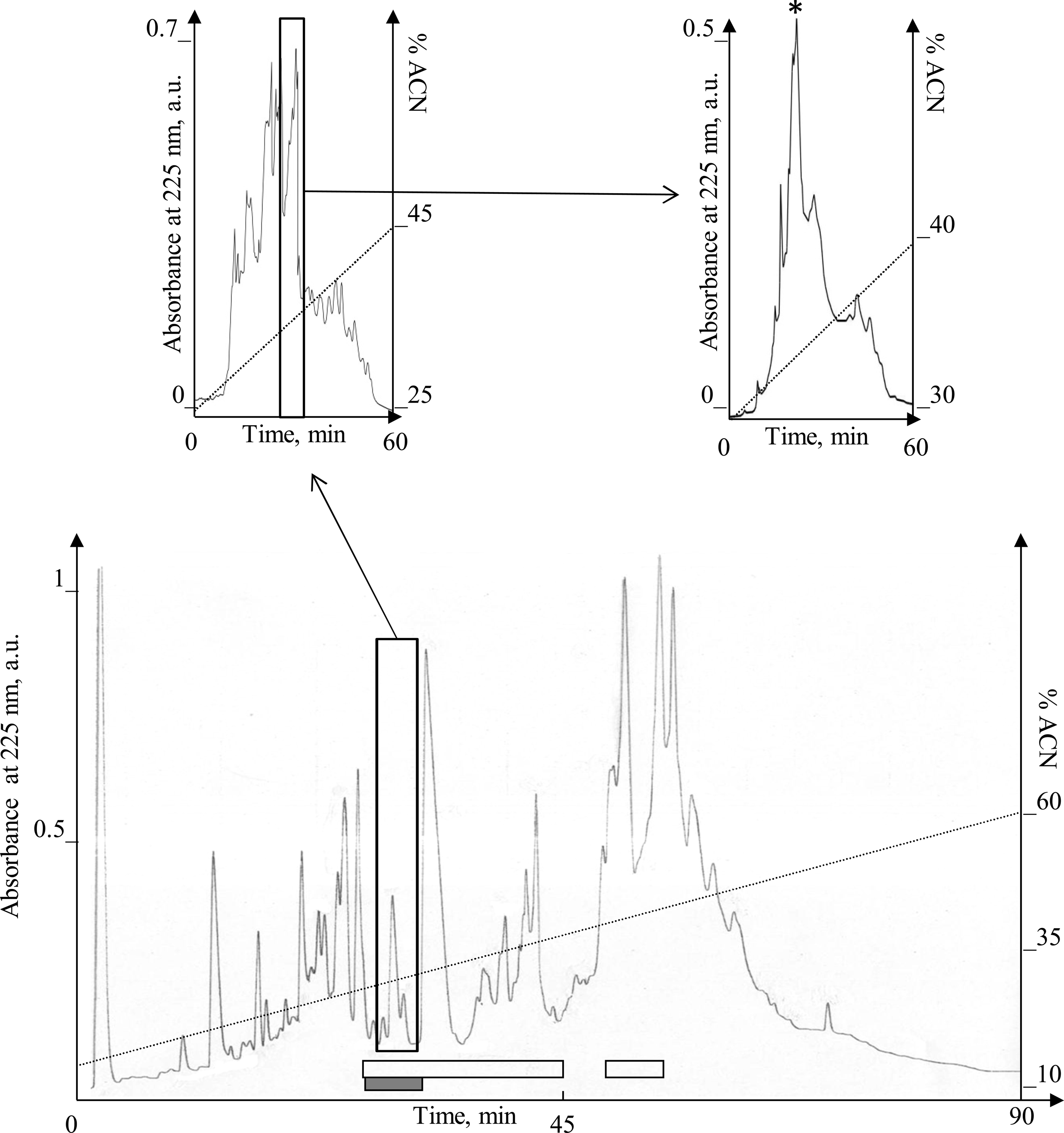

Supplement: Figure S1 — Alvinellacin purification and molecular identification. Material eluting at 60% acetonitrile (ACN) upon solid phase extraction was loaded onto a C18 column (250×4 mm, Vydac). Elution was performed with a linear gradient of acetonitrile in acidified water (dotted line), and absorbance was monitored at 225 nm. Each individually collected fraction was tested for its antimicrobial activity (white bar) and its immunoreactivity to the alvinellacin Ab by DIA (grey bar). Fractions containing antimicrobially active alvinellacin were further purified by two additional RP-HPLC purification steps. Asterisk shows the active final fraction containing alvinellacin. (TIF) [file pone.0095737.s001.tif]

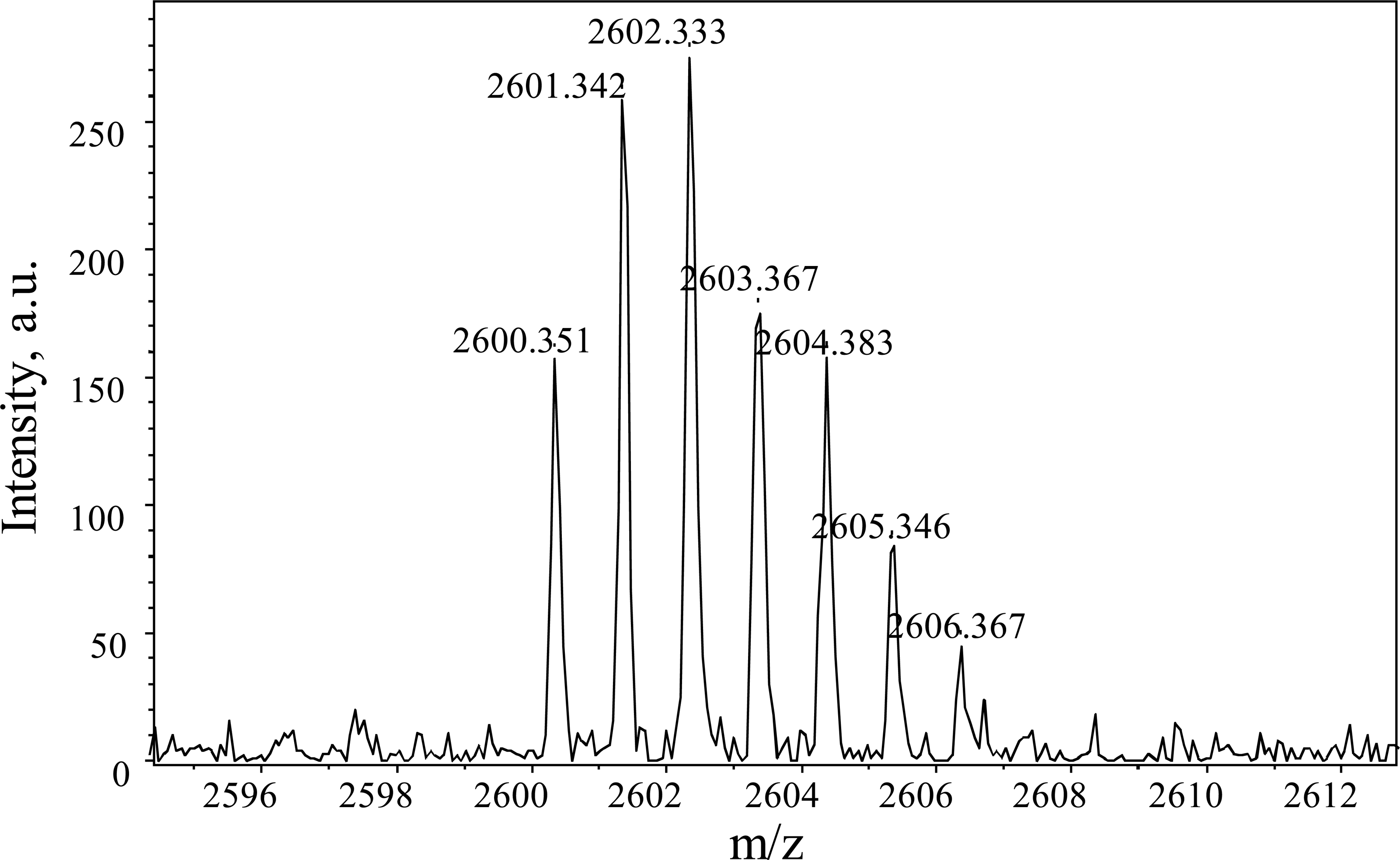

Supplement: Figure S2 — MS spectrum of native alvinellacin. Analysis of purified alvinellacin by MALDI TOF-MS shows a m/z value of 2,600.35 MH+ which perfectly matches the theoretical mass of the peptide including two disulfide bonds. (TIF) [file pone.0095737.s002.tif]

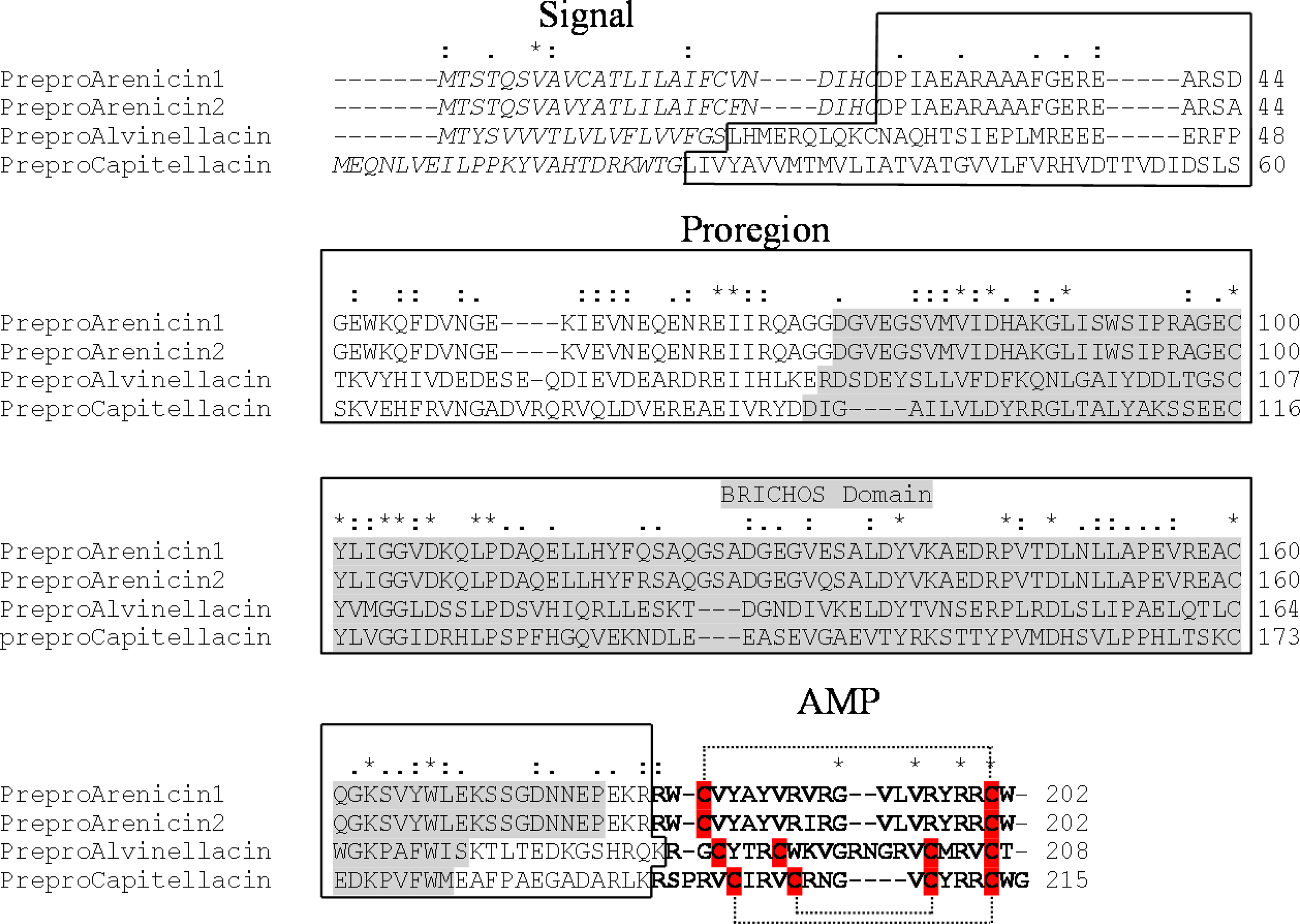

Supplement: Figure S3 — Sequence alignments of the precursors of alvinellacin, capitellacin, and two arenicin isoforms. (TIF) [file pone.0095737.s003.tif]

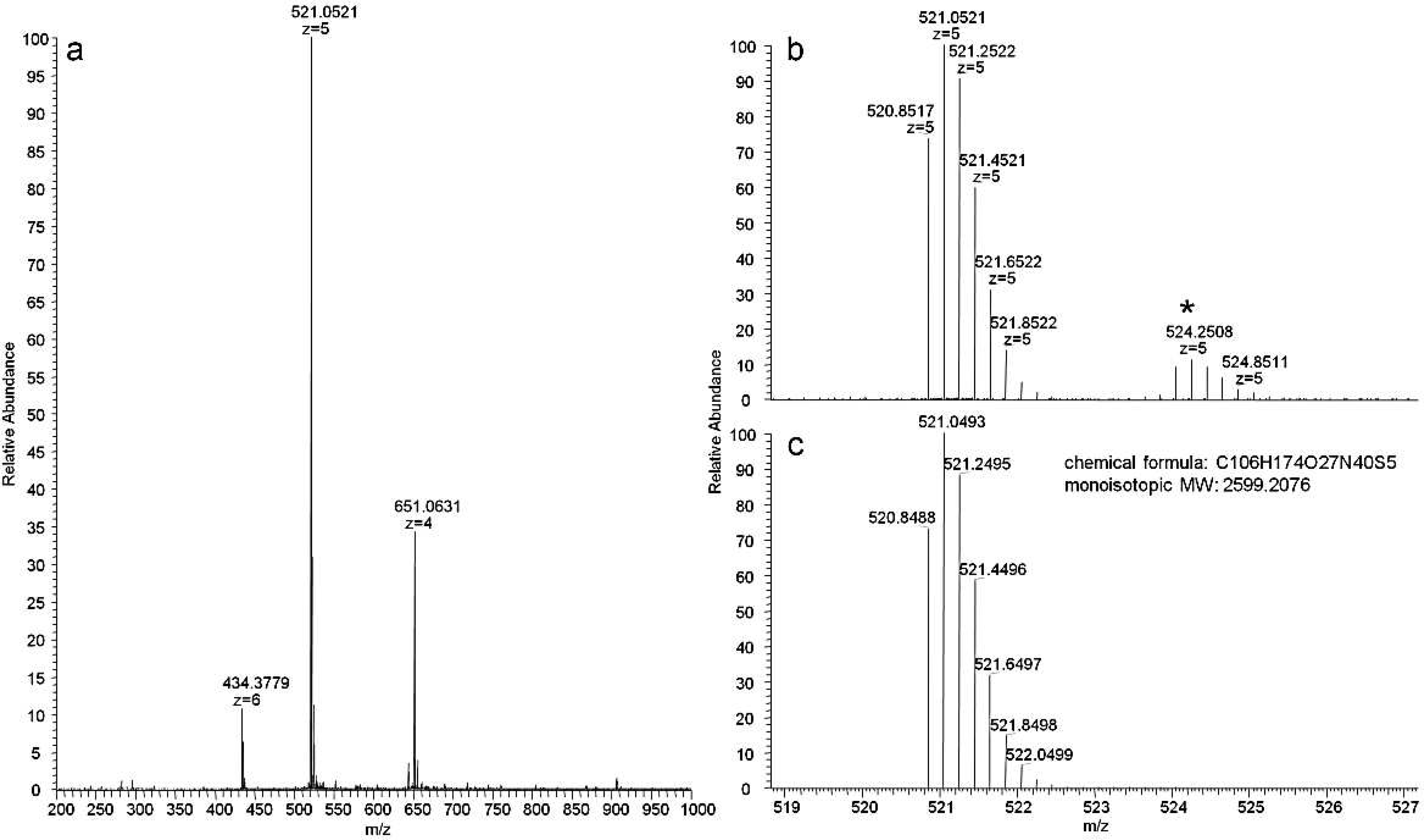

Supplement: Figure S4 — Intact protein MS spectrum of alvinellacin measured by nanoESI-Orbitrap MS. (A) Full range MS survey spectrum. (B) Zoom-in of the [M+5H]5+ charge state species in a. A small species (indicated as asterisk) found next to the major component was identified as the methionine oxidation product of alvinellacin. The experimentally determined monoisotopic MW of alvinellacin was 2,599.2221 Da. (C) Display of theoretical MW (2,599.2067 Da) of alvinellacin and its isotope distribution at charge state 5. The results indicated that all four cysteines are involved in the formation of disulfide bonds. (TIF) [file pone.0095737.s004.tif]

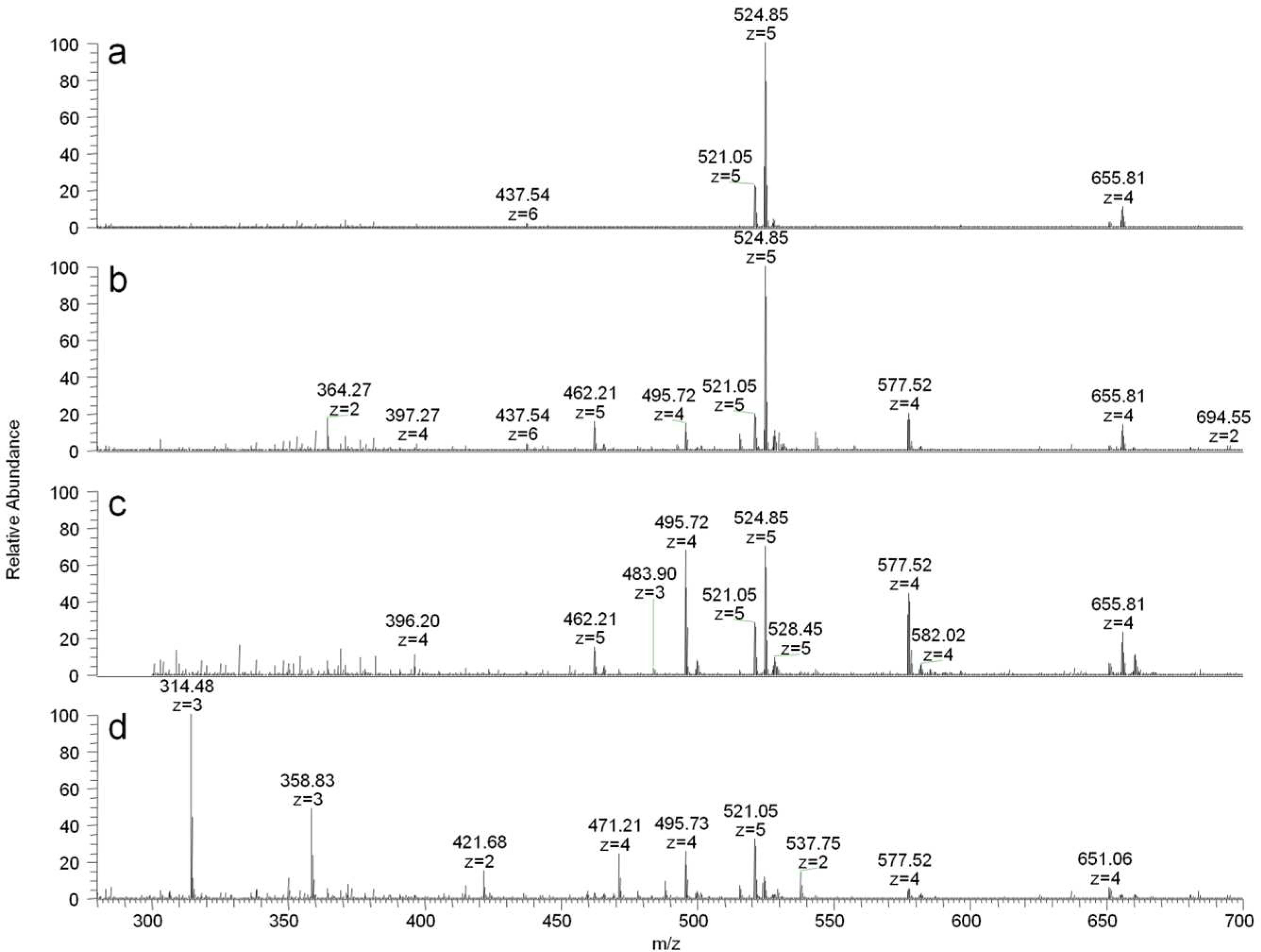

Supplement: Figure S5 — Time-course analysis of the proteolytic cleavage of alvinellacin. The products of alvinellacin digestion were analyzed by nanoESI-Orbitrap MS. (A) Peptide MS survey spectra of alvinellacin digested with Lys-C at 35°C (overnight). (B) Subsequent digestion of the Lys-C-digest with trypsin after 30 min; (C) after 2 h; (D) after 18 h at 37°C. The identities of the peptides are summarized in Table S2. (TIF) [file pone.0095737.s005.tif]

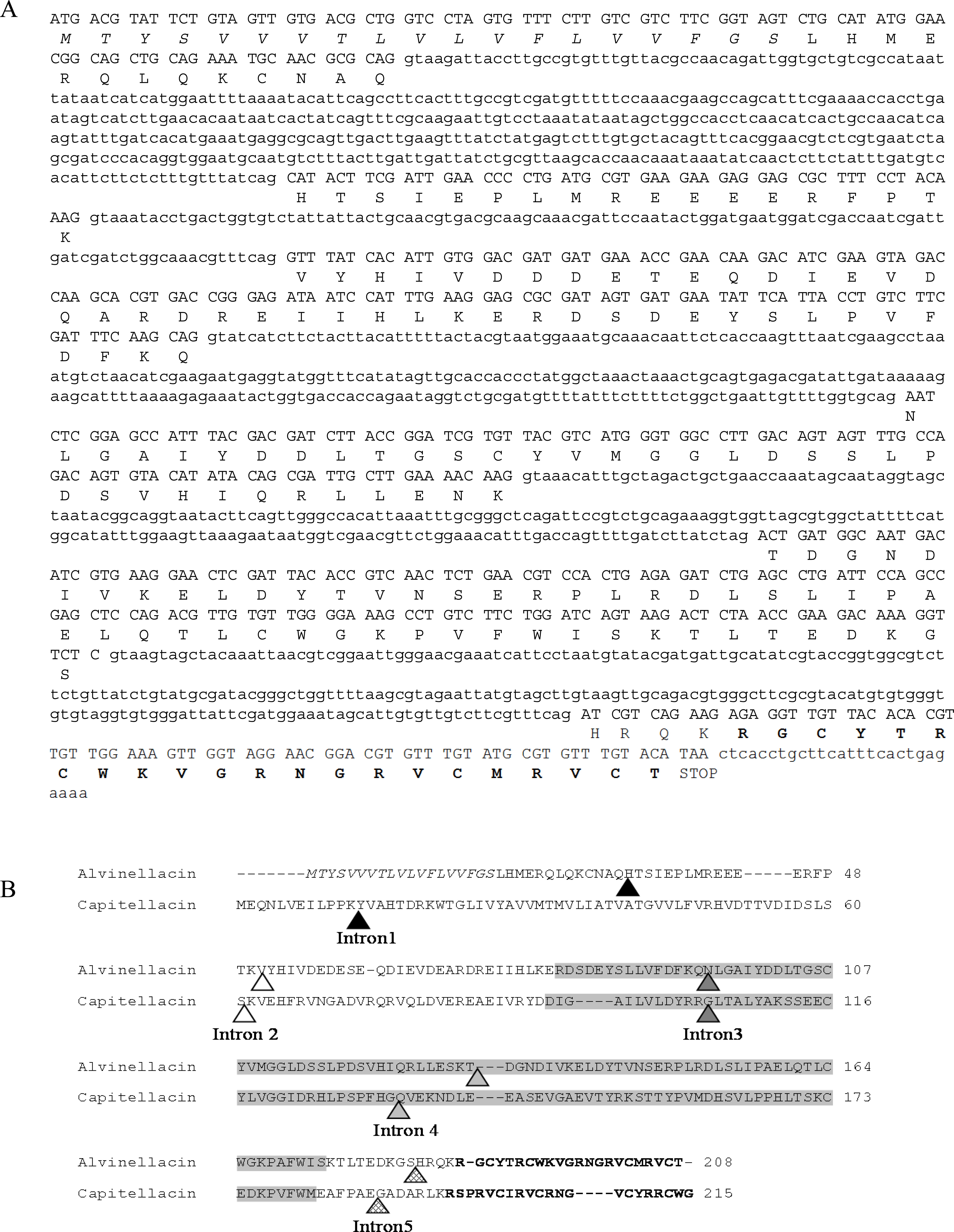

Supplement: Figure S6 — Alvinellacin and capitellacin gene structures. (A) As opposed to CDS (648 bp), the alvinellacin gene is rather long (1949 bp from the initial methionine to the stop codon) with a 5 introns/6 exons structure and a first large intron of 442 bp. Introns are all inserted in phase 0 with the exception of the last one in phase 1. (B) Alignment of the translated regions of the alvinellacin and capitellacin genes. The intron splicing positions (triangles) are nearly conserved. The BRICHOS domains are shaded and the AMP sequences are in bold type. (TIF) [file pone.0095737.s006.tif]
