# Supplementary material for: Characterization and Function of the First Antibiotic Isolated from a Vent Organism: The Extremophile Metazoan Alvinella pompejana
Source: PLoS One. 2014 Apr 28;9(4):e95737. doi: 10.1371/journal.pone.0095737 (PMC4002450; doi:10.1371/journal.pone.0095737)
Supplement: Material and Methods S1 — (DOCX) [file pone.0095737.s007.docx]

**Supplementary Material and Methods**

**S1: Microorganisms**

The bacterial strains used were: *Bacillus megaterium* (ATCC 14581), *Escherichia coli* K-12 strain D31, an ampicillin- and streptomycin-resistant strain [1], and *Staphylococcus aureus* (Institut Pasteur, Lille). The pure hydrothermal strains were *Thermococcus fumicolans*, *Thermococcus hydrothermalis*, *Pyrococcus glycovorans*, *Marinitoga camini*, *Pseudomonas sp.*, *Caminicella spirogenes*, *Vibrio diabolicus* and *Alteromonas alvinellae*. Epibionts were scraped with a thin razor from 1 cm^2^ of the tegument of *Alvinella* freshly harvested and were resuspended in 4 mL of sterile seawater. Primary enrichment cultures were obtained shipboard by adding an aliquot of epibionts or fragments of *Alvinella* tubes to 10 mL of modified SME media prepared as previously described and followed by incubation at 30 and 50°C [2] [3].

**S2: Peptide purification and identification**

Coelomic liquid (~100 mL) was collected on board, immediately frozen in liquid nitrogen and kept at -80°C until use. Liquid was centrifuged at 8,000 × g for 20 min at 4 °C. The pH value of the supernatant was brought to 3.9 with 1 M HCl. Centrifugation (10,000 × g, 20 min, at 4 °C) then was performed to clarify the supernatants, which were loaded onto Sep-Pak C18 Vac cartridges (Waters). Elution steps were performed with 2 and 60% acetonitrile (ACN) in acidified water. The prepurified fractions were then concentrated, reconstituted in pure water, and tested for antimicrobial activity as described below. Only the 60% ACN eluted fractions were active and submitted to purification by reversed-phase high pressure liquid chromatography (RP-HPLC). All of the following HPLC steps were carried out on a Perkin Elmer system.

Step 1—Aliquots of the 60% Sep-Pak fractions were subjected to RP-HPLC on a Sephasyl C18 column (250 × 4.1 mm, model 218TP54, Vydac). Elution was performed with a linear gradient of 2–62% ACN in acidified water over 90 min at a flow rate of 1 mL/min. The fractions corresponding to absorbance peaks were collected in polypropylene tubes, dried, reconstituted in water, and tested for antimicrobial activity.

Step 2—The active fractions were loaded further onto a C18 column (250 × 2.1 mm, model 218TP52, Vydac) with a gradient consisting of 2–25% ACN in acidified water for 10 min and 25–45% ACN for 60 min at a flow rate of 0.2 mL/min. The fractions were collected and treated as above.

Step 3—One additional step was performed on a Narrowbore C18 reversed-phase column (150 × 2 mm, Waters) with a gradient consisting of 30–40% ACN in acidified water for 60 min.

Purity was assessed by mass spectrometry analyses (DE STR PRO; Applied Biosystems) and homogeneous material was subjected to protein sequencing via Edman degradation (pulse liquid automatic peptide sequenator, Beckman Coulter).

**S3: Renaturation and NMR spectroscopy**

Alvinellacin was purchased from Biosyntan GmbH (Germany) in its linearized, reduced form. The freeze-dried peptide sample was reconstituted in 10 mM hydrogen chloride and renaturated at 4°C in 0.8 M guanidinium chloride, 40 mM Tris, 3.2 mM reduced glutathion, 0.32 mM oxidized glutathione (pH 8.5) at a peptide concentration of 0.16 mg/mL for 24 h. After renaturation, salts were removed using a Sep-Pak® Vac 6cc (500 mg) tC18 cartridge (Waters). The bound sample was eluted in 84% (v/v) ACN in acidified (0.1% (v/v) trifluoric acid) water and freeze dried. Finally, alvinellacin was purified by RP-HPLC using a C18-column and a continuous ACN gradient in acidified (0.1% (v/v) trifluoric acid) water. The fraction containing alvinellacin was verified by mass spectrometry and freeze dried. Finally, the renaturated alvinellacin peptide was reconstituted in distilled water supplied with 1% (v/v) d4-acetic acid, 10% (v/v) D2O yielding a final concentration of 2 mM peptide. The sample was transferred into a symmetrical matched microtube (Shigemi, Inc.). NMR measurements were performed on a Bruker Avance III 800 MHz spectrometer. Sequence-specific backbone resonance assignments of alvinellacin were established using two-dimensional total correlation spectroscopy experiments with different mixing times (60, 100 and 120 ms). Distance restraints were obtained from two-dimensional nuclear Overhauser effect (NOE) spectroscopy experiments with different mixing times (80, 100, 120, 150, 180 and 200 ms). The chemical shift data were deposited in the University of Wisconsin Biological Magnetic Resonance Bank database under the accession number 18085. All spectra were processed with the program NMRPipe [4] and analyzed with the program NMRView [5].

Models of the three dimensional structure of capitellacin were generated using the solution NMR structure of alvinellacin as template. According to the sequence alignment, amino-acid residues of capitellacin were exchanged in the template. Deletions in the molecule were modeled using a database search approach included in the software package WHATIF [6].

Structure calculation—Structure calculations were performed using the program CYANA [7]. The calculations were based on 104 inter-proton constraints. Distance restraints derived from the NMR spectra were calibrated using an r^6 function. Two disulfide bonds were defined as six distance restraints ranges as follows: 2.0 ≤ d(Siγ, Sjγ) ≤ 2.1 Å; 3.0 ≤ d(Ciβ, Sjγ) ≤ 3.1 Å; 3.0 ≤ d(Siγ, Cjβ,) ≤ 3.1 Å. 100 structures were calculated and sorted according to the lowest target-function value. The 10 best structures were selected as the final structural ensemble and were deposited (PDB accession code 2LLR).

**S4 Assignment of disulfide bridges in alvinellacin by Mass Spectrometry**

Lys-C and trypsin were purchased from Promega (Madison, MI). Tris-buffer and EDTA were purchased from Carl Roth GmbH (Karlsruhe, Germany). Acetonitril Optima® LC/MS was from Fischer Scientific and formic acid was from Fluka. Water, used for all experiments, was purified with an arium®611VF System from Sartorius (Göttingen, Germany). A stock solution of 10 µg of oxidized synthetic alvinellacin (0.01 mg/mL in 0.01% TFA) was used for this study.

Alvinellacin was diluted with digestion buffer (25 mM Tris-HCl, 1 mM EDTA, pH 8.5) to a final concentration of 2 pmol/µL. For Lys-C digestion, the endoproteinase Lys-C (1:400 w/w) was added and incubated for 18 h at 35°C. For sequential Lys-C and tryptic digestion, trypsin (1:400 w/w) was added to the Lys-C digestion to digest alvinellacin for further 18 h at 37°C. During the digestion, aliquots of 10 µL were taken from the digest at different time points in order to monitor the enzyme digestion efficiency. In all cases, digestion was stopped by addition of 1% formic acid.

ZipTip pipette tips (Millipore, Billerica, MA) were used for desalting and concentrating the peptides prior to nanoESI-Orbitrap MS analysis according to the producer guidelines. The elution was performed with 5 μL 0.1 % formic acid in 50 % ACN.

MS experiments were performed on an offline nanoESI-LTQ Orbitrap Velos mass spectrometer with ETD option (Thermo Fisher Scientific, San Jose, CA) MS was operated either with FT-MS mode for full-MS survey or CID-MS2/ETD-MS2 for MSMS fragmentation. Spectrum interpretation and disulfide bridge assignment were performed manually.

**REFERENCES**

1. Boman HG, Nilsson-Faye I, Paul K and Rasmuson T, Jr. (1974) Insect immunity. I. Characteristics of an inducible cell-free antibacterial reaction in hemolymph of *Samia cynthia* pupae. Infect Immun 10: 136-145.

2. Stetter KO, Konig H and Stackebrandt E (1983) *Pyrodictium* gen. nov., a New Genus of Submarine Disc-Shaped Sulphur Reducing Archaebacteria Growing Optimally at 105 degrees C. Syst Appl Microbiol 4: 535-551.

3. Vetriani C, Speck MD, Ellor SV, Lutz RA and Starovoytov V (2004) *Thermovibrio ammonificans* sp. nov., a thermophilic, chemolithotrophic, nitrate-ammonifying bacterium from deep-sea hydrothermal vents. Int J Syst Evol Microbiol 54: 175-181.

4. Delaglio F, Grzesiek S, Vuister GW, Zhu G, Pfeifer J, et al. (1995) NMRPipe: a multidimensional spectral processing system based on UNIX pipes. J Biomol NMR 6: 277-293.

5. Johnson BA (2004) Using NMRView to visualize and analyze the NMR spectra of macromolecules. Methods Mol Biol 278: 313-352.

6. Vriend G (1990) WHAT IF: a molecular modeling and drug design program. J Mol Graph 8: 52-56.

7. Güntert P (2004) Automated NMR structure calculation with CYANA. Methods Mol Biol 278: 353-378.
