# Supplementary material for: Characterization and Function of the First Antibiotic Isolated from a Vent Organism: The Extremophile Metazoan Alvinella pompejana
Source: PLoS One. 2014 Apr 28;9(4):e95737. doi: 10.1371/journal.pone.0095737 (PMC4002450; doi:10.1371/journal.pone.0095737)
Supplement: Table S1 — Structural statistics for the 10 best structures of alvinellacin showing the lowest target functions. None of the distance constraints was violated by more than 0.5 Å in any structure. (DOCX) [file pone.0095737.s008.docx]

**Table S1.** Structural statistics for the 10 best structures of alvinellacin showing the lowest target functions. None of the distance constraints was violated by more than 0.5 Å in any structure.

| ***Distance restraints*** | *Number* |
| --- | --- |
| Intraresidual (\|i-j\| = 0) | 38 |
| Sequential (\|i-j\| = 1) | 40 |
| Medium range (2 ≤ \|i-j\| ≤ 4) | 0 |
| Long range (\|i-j\| ≥ 5) | 14 |
| Disulfide bonds (included) | 12 |
| Total | 104 |
| ***Pairwise rmsd*** | |
| Mean global backbone rmsd to mean* | 0.49 ± 0.20 Å |
| Mean global heavy rmsd to mean* | 1.47 ± 0.20 Å |
| ***Ramachandran plot*** |  |
| Most favored regions | 73.5 % |
| Additional allowed regions | 18.8 % |
| Generously allowed regions | 6.5 % |
| Disallowed regions | 1.2 % |

* Residues considered: 4-9, 14-19
