# Supplementary material for: Characterization and Function of the First Antibiotic Isolated from a Vent Organism: The Extremophile Metazoan Alvinella pompejana
Source: PLoS One. 2014 Apr 28;9(4):e95737. doi: 10.1371/journal.pone.0095737 (PMC4002450; doi:10.1371/journal.pone.0095737)
Supplement: Table S2 — Disulfide-connected peptide fragments of alvinellacin observed after proteolytic cleavage. Peptides with oxidized cysteines were successively digested using the proteases Lys-C and trypsin. The resulting peptides were analyzed by offline nanoESI-Orbitrap MS/MS as shown in Figure S2. The results unambiguously indicated two disulfide linkages between C1–C4 and C2–C3. (DOCX) [file pone.0095737.s009.docx]

**Table S2.** Disulfide-connected peptide fragments of alvinellacin observed after proteolytic cleavage. Peptides with oxidized cysteines were successively digested using the proteases Lys-C and trypsin. The resulting peptides were analyzed by offline nanoESI-Orbitrap MS/MS as shown in Figure S2. The results unambiguously indicated two disulfide linkages between C1-C4 and C2-C3.

| **Enzyme** | **Observed disulfide-linked peptides** | **Observed ions** | **Disulfide-linked peptide Mw**  **(Da, monoisotopic)** | |
| --- | --- | --- | --- | --- |
|  |  |  | **theor.** | **exp. ± SD** |
| Lys-C | RGC_1_YTRC_2_WKVGRNGRVC_3_MRVC_4_T  (with 2 internal disulfide bonds) | 520.849 (5+)  650.810 (4+) | 2599.2076 | 2599.210 ± 0.002 |
|  | RGC_1_YTRC_2_WK  \| \|  VGRNGRVC_3_MRVC_4_T  or  RGC_1_YTRC_2_WK  \|____\|____  \| \|  VGRNGRVC_3_MRVC_4_T | 524.448 (5+)  655.308 (4+) | 2617.2182 | 2617.203 ± 0.001 |
| trypsin | RGC_1_YTRC_2_WK  \|____\|____  \| \|  NGRVC_3_MRVC_4_T | 462.014 (5+)  577.265 (4+) | 2305.0272 | 2305.032 ± 0.002 |
|  | RGC_1_YTRC_2_WK  \|____\|____  \| \|  VC_3_MRVC_4_T | 495.474 (4+)  660.295 (3+) | 1977.8617 | 1977.865 ± 0.003 |
|  | RGC_1_YTR  \|  VC_4_T | 358.833 (3+)  537.746 (2+) | 1073.4746 | 1073.477 ± 0.001 |
|  | C_2_WK  \|  VC_3_MR | 314.477 (3+)  471.213 (2+) | 940.4082 | 940.410 ± 0.002 |
